# Photooxidation-Guided Ultrastructural Identification and Analysis of Cells in Neuronal Tissue Labeled with Green Fluorescent Protein

**DOI:** 10.1371/journal.pone.0064764

**Published:** 2013-05-31

**Authors:** Heinz Horstmann, Mariya Vasileva, Thomas Kuner

**Affiliations:** Institute of Anatomy and Cell Biology, Heidelberg University, Heidelberg, Germany; Virginia Tech Carilion Research Institute, United States of America

## Abstract

The ultrastructural characterization of neuronal compartments in intact tissue labeled with green fluorescent protein (GFP) remains a frequently encountered challenge, despite work establishing photooxidation of GFP in cultured cells. However, most applications require the detection of GFP or GFP fusion proteins expressed in intact tissue. Here, we report that illumination of GFP variants in oxygen-enriched environment reliably generated electron-dense 3,3′-diaminobenzidine (DAB) precipitates in slices from rat brain. The method is applicable to GFP variants tagged to presynaptic proteins as well as to soluble GFP in various brain regions. Serial section scanning electron microscopy was used to examine genetically labeled presynaptic terminals at high resolution and to generate three-dimensional representations of the synapses. Thus, we introduce a generally applicable correlative approach for the identification of presynaptic terminals genetically labeled with green fluorescent proteins in tissue slices and their ultrastructural characterization.

## Introduction

Green fluorescent protein (GFP) and its derivatives have been intensely used as genetically encoded reporters for intracellular protein expression or for the examination of protein-protein interactions at the resolution of fluorescence microscopy [1], [2]. However, the direct detection of genetically labeled cells at the resolution of electron microscopy remains challenging, yet is often required to complement observations made on larger scales. Approaches of correlative microscopy in general provide one solution to identify fluorescently labeled structures and subsequently examine them using the electron microscope (EM) [3]–[7]. One strategy relies on photooxidation of genetically encoded probes resulting in a 3,3′-diaminobenzidine (DAB) electron-dense product that can be visualized with the EM [8]–[10]. However, these approaches require high local concentrations of GFP (e.g. Golgi-resident enzyme N- acetylgalactoseaminyltransferase-2 fused to EGFP [8]) and have only been demonstrated in cultured cells. Alternatively, miniSOG could be used, a protein reported to be a more efficient singlet oxygen generator (e.g. Connexin fused to miniSOG) [9]. However, due to the widely spread use of GFP as a fluorescent protein-tag, GFP-positive cells may need to be analyzed on the ultrastructural level. This is challenging because of the limited ability of GFP to produce singlet oxygen [11]. Therefore, a method capable of directly visualizing fluorescent proteins in intact tissue via photooxidation would be highly desirable. Correlation of fluorescence with electron microscopy often works when looking at the cell soma but may be difficult when small compartments such as presynaptic nerve terminals need to be studied.

To achieve this, we used recombinant adeno-associated viruses to express EGFP or EGFP fusion proteins (synaptophysin fused with two pHluorins (pH-sensitive GFP version [12]), synaptophysin-EGFP, EGFP-synapsin I) in giant glutamatergic synapses in the rat auditory brainstem [13] and thalamus [14]. We show that illumination of presynaptic terminals expressing EGFP or EGFP fusion proteins under carefully controlled conditions generates an amount of reactive oxygen species sufficient to oxidize DAB into electron-dense precipitates, which can be detected in the EM. The precipitates can be visualized with light microscopy and used for targeted trimming of the tissue block in preparation for EM. We used serial section scanning electron microscopy (S^3^EM [15]) to examine and to reconstruct genetically labeled presynaptic terminals.

## Results

### Fluorescence Signals Correlated to DAB Precipitates in Calyces Expressing Synaptophysin-2-pHluorin

**Figure 1:**
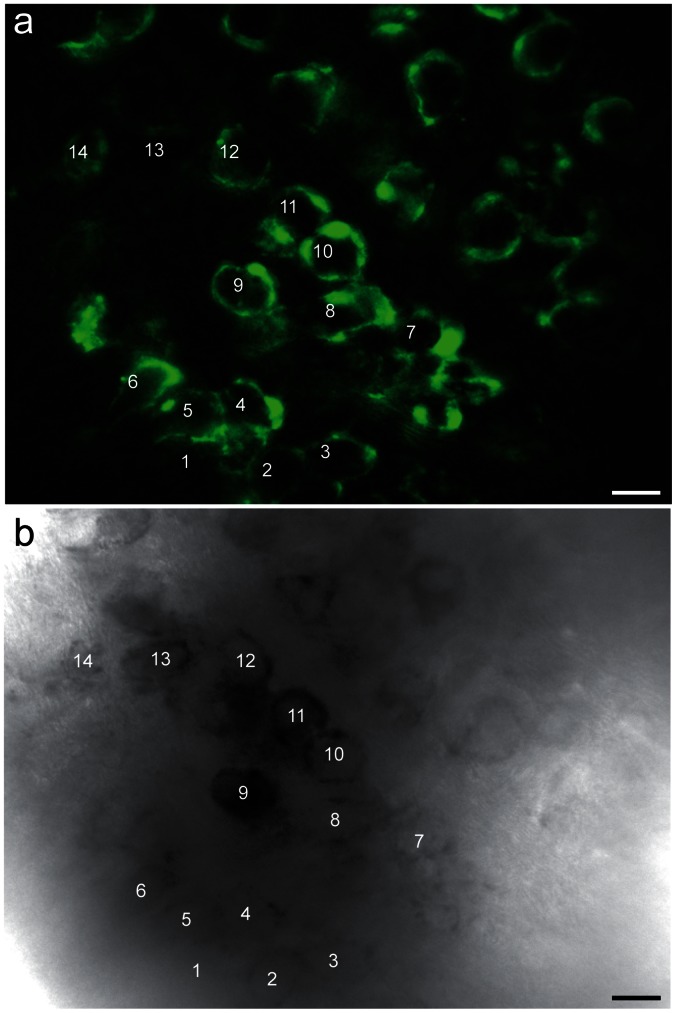
Correlation of synaptophysin-2-pHluorin fluorescence and photooxidation product. (a) MNTB with calyces expressing synaptophysin-2-pHluorin (wide-field fluorescence microscopy). (b) Corresponding region after photooxidation imaged with bright-field light microscopy. The region of interest was illuminated for 15 min with λ = 488 nm. At the end of this period the fluorescence signal disappeared and dark granular DAB precipitates appeared within the infected calyces. The minor differences of the pattern revealed by the precipitate compared to the fluorescent image may be due to different focal planes of the images. Scale bars, 20 µm.

Synaptophysin-2-pHluorin was expressed in the calyx of Held using targeted viral gene transfer. Circular patterns of labeled vesicle clusters typical for the adult calyx [16] were amply present in the medial nucleus of the trapezoid body (MNTB) (Fig. 1a). Focal illumination under controlled oxidative conditions generated free oxygen radicals and formed DAB precipitates (see Materials & Methods) that were readily detectable in the light microscope after 15 min illumination with λ = 488 nm (Fig. 1b). The DAB precipitates formed gradually with increasing illumination duration and were located exclusively within the illuminated region containing calyces expressing synaptophysin-2-pHluorin. The intensity of the DAB precipitate differed between individual calyces because the photooxidation is proportional to the level of overexpressed protein [8].

**Figure 2:**
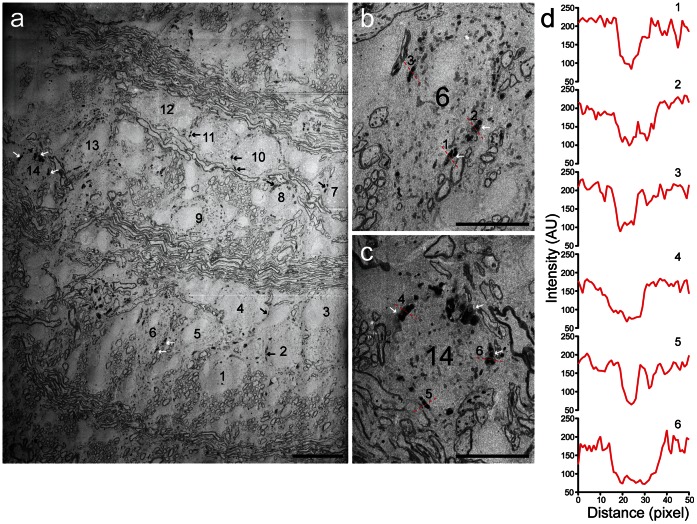
Photooxidized DAB product can be detected with the EM. (a) Electron-dense DAB precipitates (arrows) in a low-magnification electron micrograph taken with the SEM. Numbers indicate the principal cells corresponding to those shown in Figure 1. (b) Digital magnification of cell #6. (c) Digital magnification of cell #14. (d) Line profiles show drop in the signal intensity, measured in arbitrary units (AU), across photooxidized presynaptic compartments, dotted red lines in (b,c). Myelinated axons are also visible as structures of high contrast. Scale bars, (a) 20 µm; (b, c) 10 µm.

For electron microscopy the tissue was trimmed and embedded in epoxy. The tissue block was serially sectioned to produce ultrathin sections of the region of interest [15]. The electron-dense material of the DAB precipitate could be readily identified in tissue from the MNTB allowing us to identify the synaptophysin-2-pHluorin overexpressing presynaptic terminals by scanning electron microscopy. The calyces containing the DAB precipitate appeared darker than the surrounding tissue (Fig. 2). A direct correlation between light and electron microscopy at low magnification revealed MNTB principal cells (numbering as in Fig. 1) nested within axonal bundles. Along the circumference of the MNTB principal cells dark spots were visible (arrows) representing photooxidized DAB within presynaptic terminals containing synaptophysin-2-pHluorin. The photooxidation product is more clearly presented in the insets showing two principal cells with calyces expressing the fusion protein (Fig. 2b–c). Probe lines prove the presence of dark DAB precipitate in the calyceal compartments but not in the soma of the postsynaptic cell or in other areas outside the synaptic structure (Fig. 2d).

The formation of the DAB precipitate depends on the concentration of the overexpressed protein in the presynaptic terminal. The amount of viral protein overexpression can vary between individual cells, giving rise to a corresponding intensity range of the DAB precipitate. Furthermore, due to the high infection efficiency it is difficult to find calyces lacking the expressed fluorescent protein within the MNTB corresponding to the transduced cochlear nucleus. Therefore, we used MNTB regions not containing calyces overexpressing GFP for control measurements. When these regions were illuminated with our standard protocol in the presence of DAB, dark precipitates were not observed (Fig. S1). The signal intensity of mitochondria in non-infected terminals is comparable to that of mitochondria in the principal cell (Fig. S1d,e). Images were taken prior to counterstaining with saturated solutions of uranyl acetate and lead citrate.

Additionally, we examined neighboring cells at the rim of the illumination region (Fig. S2). The region of interest was selected in such a way that one cell was inside the illuminated region (Fig. S2a, white circle), while the neighboring one remained outside. Dark DAB precipitates (black arrows) were observed along the circumference of the postsynaptic cell outlining the photooxidized fluorescent protein in the presynaptic terminal. DAB precipitates did not form within the presynaptic terminal that remained outside the illuminated region.

### Three-dimensional Reconstructions of Identified Presynaptic Terminals

Upon identification of photooxidized DAB in the presynaptic terminals, we prepared silicon wafer strips containing a ribbon of serial ultrathin sections [15]. To allow unequivocal detection of the DAB precipitation sections were imaged before and after counterstaining (Fig. S3).

**Figure 3:**
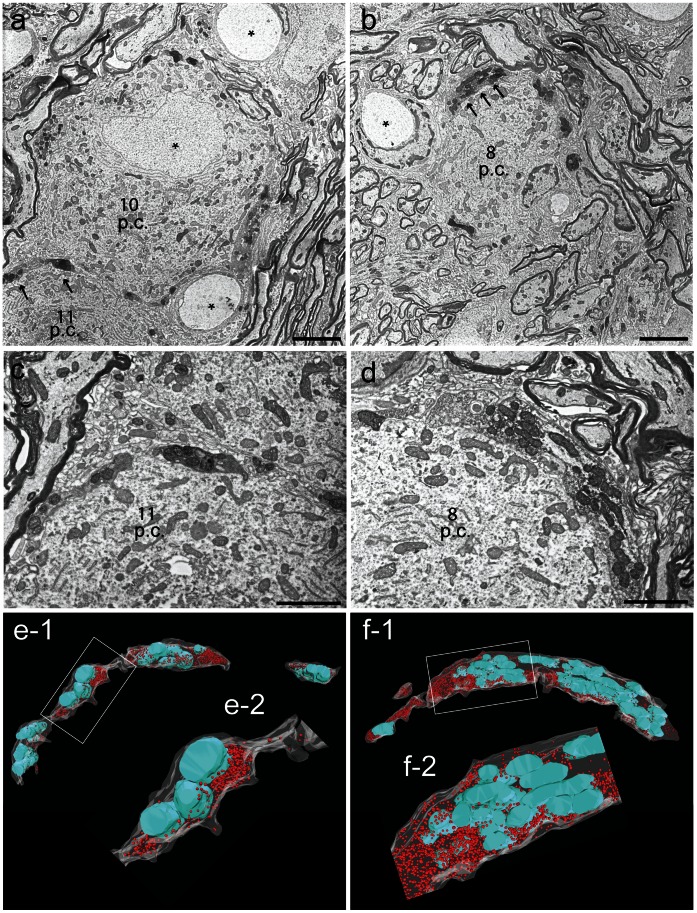
Photooxidized calyces at higher magnification. (a) Cells #10/11 and (b) Cell #8 shown in Figure 1 imaged at low magnification of Principal cell nuclei are denoted with (*). (c,d) Regions pointed out with white arrows in (a,b) shown at higher resolution. (e-1,f-1) Corresponding 3D reconstructions generated from 17/16 consecutive sections (35 nm thickness). (e-2,f-2) Digitally magnified segments from the presynaptic compartments, corresponding to the rectangular shapes in (e-1) and (f-1). Presynaptic segment – white, synaptic vesicles – red spheres, mitochondria – cyan, p.c. MNTB principal cell. Scale bars, (a, b) 5 µm; (c, d) 2 µm.

Photooxidized DAB precipitates could be most conveniently detected within the presynaptic terminal at low resolution. Therefore, images of MNTB principal cells (#10,11 and #8 in Fig. 2) innervated by calyces containing synaptophysin-2-pHluorin imaged were first acquired at low resolution after counterstaining (Fig. 3a and b, arrows). Upon identifying photooxidized presynaptic compartments, images from consecutive sections were obtained with higher resolution to reveal the presynaptic architecture (Fig. 3c and d). The examined segments contain large numbers of mitochondria and synaptic vesicles. The dark mitochondria within the presynaptic compartment can also be used to recognize the infected cells for correlative microscopy [4]. To exclude the possibility that the described manipulations lead to ultrastructural alterations and malformations within the presynaptic terminals, the segments were followed through 17 (Fig. 3c) and 16 (Fig. 3d) consecutive sections and three-dimensional reconstructions were generated (Fig. 3e-1 and f-1). Synaptic vesicles (red spheres) and mitochondria (cyan) were found within the entire reconstructed volume and formed clusters close to the presynaptic release face (Fig. 3e-2 and f-2). An additional presynaptic compartment and a 3D reconstruction are shown in Figure S4. The consecutive sections used for the generation of the 3D reconstruction are presented in Movies S1–3.

**Figure 4:**
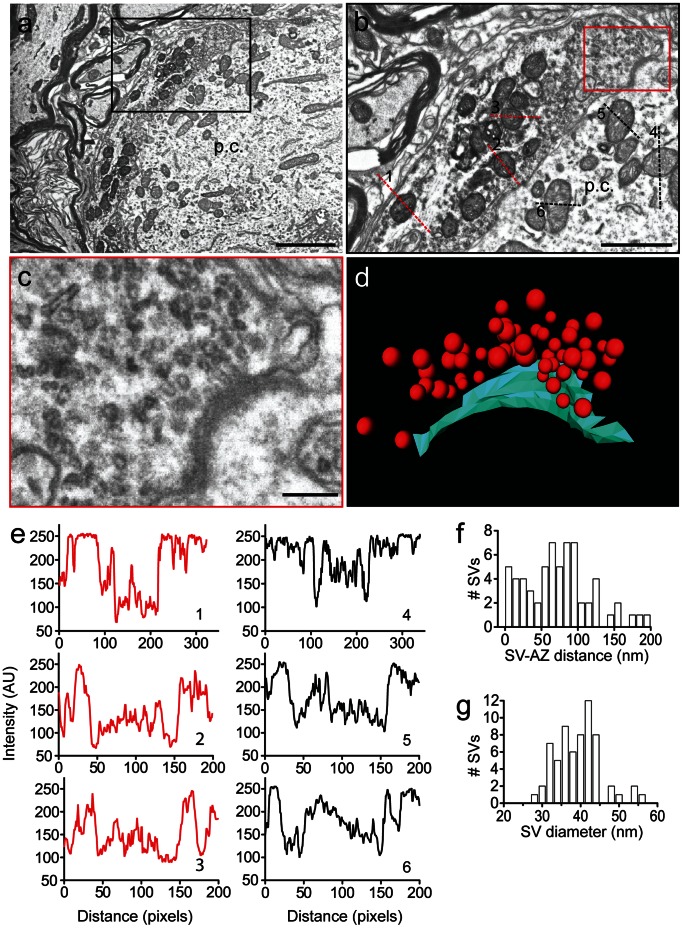
High magnification of a photooxidized calyx and active zone reconstruction. (a) Magnified view of calyx #8. Dark mitochondria and generally increased density reflecting photooxidized DAB precipitate. (b) Digital magnification of the presynaptic terminal depicted in (a). The magnified region corresponds to the rectangular shape in (a). (c) Single active zone with the associated vesicle cluster (magnification of the rectangular region in (b)). p.c.: MNTB principal cell. (d) 3D reconstruction of the active zone presented in (c), synaptic vesicles – red spheres, active zone surface area - cyan. (e) Intensity line profiles through the mitochondria shown in (b). Red dotted lines – mitochondria in the presynaptic terminal (1–3), black dotted lines – mitochondria in the principal cell (4–5). (f) Histogram of the vesicle distribution within the cluster relative to the plasma membrane at the surface of the active zone. (g) Histogram of the synaptic vesicle diameter. Average diameter was 39 nm. Scale bars, (a) 2 µm; (b) 1 µm; (c) 200 nm.

We probed further into the structural specializations of the presynaptic terminals and identified single active zones (Fig. 4). Active zones are characterized by the presence of electron dense patches of membrane – the cytomatrix of the active zone associated with postsynaptic density [17] – and synaptic vesicle clusters in close proximity (Fig. 4b–c). Since synaptophysin-2-pHluorin is targeted to the presynaptic vesicles [18], vesicles appear as dark spots within the presynaptic terminal in the EM images (Fig. 4c). Despite the intravesicular localization of pHluorin, DAB precipitate could be observed within the entire presynaptic compartment and mitochondria in the calyx expressing synaptophysin-2-pHluorin were darker than those in the principal cell (Fig. 4e). In contrast, mitochondria in non-infected terminals had comparable intensity to those in the respective postsynaptic cell (Fig. S1 d,e). Because of the fusion with a synaptic vesicle protein, some synaptophysin-2-pHluorin will also redistribute to regions of the plasma membrane including the active zone. We further examined the spatial organization of the vesicle cluster at the active zone and generated a 3D reconstruction (Fig. 4d). There were 64 vesicles within a distance of 200 nm from the active zone [19], 5 of which were within the first 10 nm and hence can be considered as docked vesicles (Fig. 4f), consistent with already published data [15], [19], [20]. The vesicles had a mean diameter of 39.32±0.74 nm (Fig. 4g), which is smaller than reported previously [19]. This discrepancy might arise from differences in the age of the animals (P20 *vs.* P9) or differences in the experimental procedure. In the 3D reconstruction the vesicles were simulated as ideal spheres with a smoothed surface. Any irregularities in the vesicle membrane and curvature were neglected. Therefore, this analysis gives only an estimate of the vesicle diameter.

The 3D reconstructions of presynaptic segments confirm that protein overexpression and the DAB photooxidation did not lead to morphological changes in the presynaptic terminal and did not introduce malformations in the general architecture of the calyx of Held. Therefore, this method can be used to compare genetically modified and control terminals in structure-function studies (Fig. S5 and data not shown).

### Other Proteins and Tissues Tested

Illumination of both cytosolic EGFP and EGFP-synapsin I fusion protein resulted in photooxidization of DAB in the calyx of Held (Figure S5). However, the EGFP DAB oxidation product is not as pronounced as in the case of EGFP-fusion proteins and is darker and easier identifiable in the low-resolution images than in the high-resolution images (Figue S5 a,c). It might be possible that the number of EGFP molecules per unit volume is small and therefore the precipitates are weak. Better results were achieved if EGFP was targeted to the presynaptic terminal using a presynaptic protein e.g. synapsin (Figure S5 b,d). From these data we conclude that EGFP, similarly to pHluorin, can be used to generate a detectable electron-dense signal by the photooxidation of DAB.

**Figure 5:**
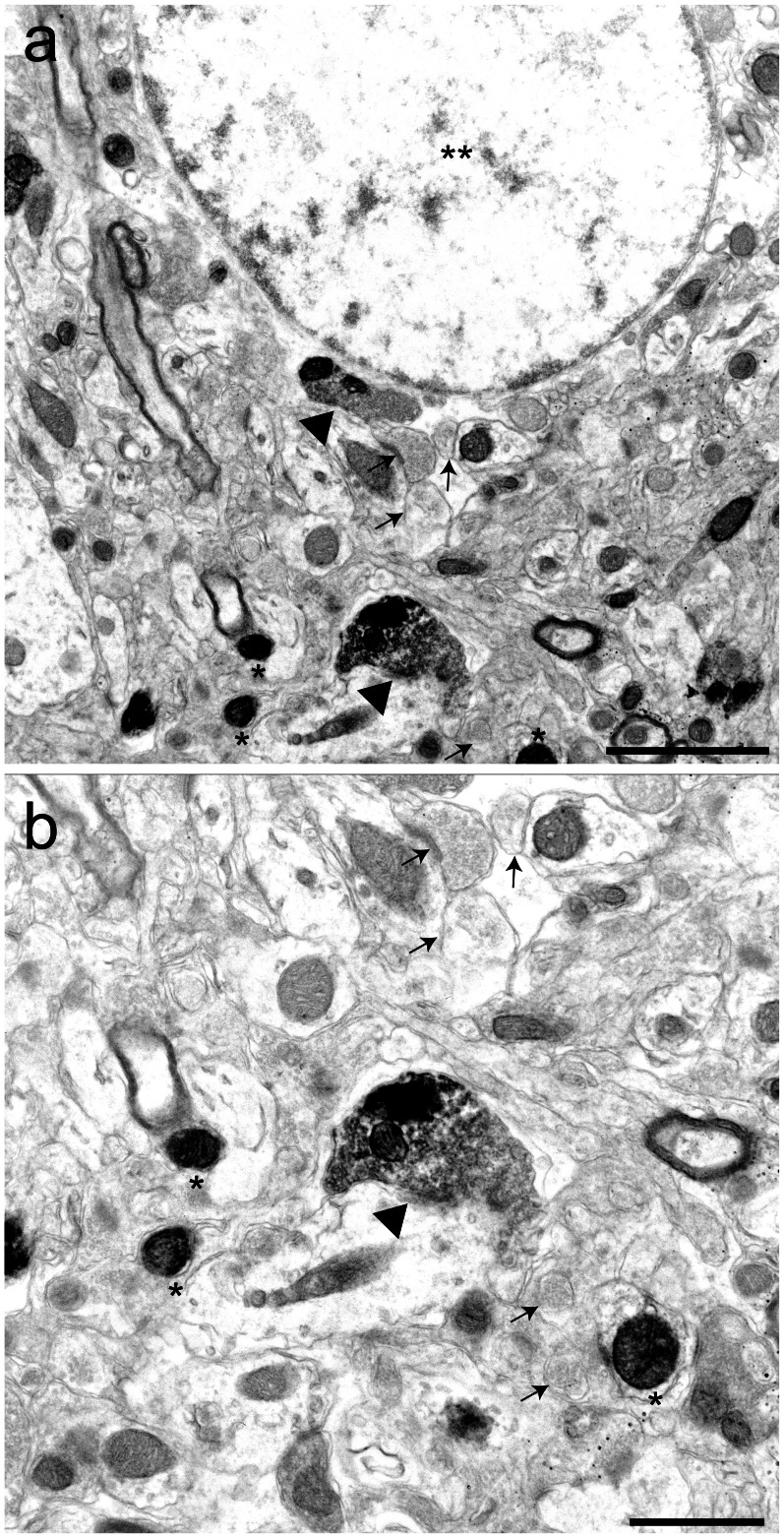
DAB precipitate in the posteromedial thalamic nucleus. Synaptophysin-EGFP was expressed in giant cortico-thalamic (L5B – POm) synapses and EGFP was used as oxygen donor for DAB photooxidation. (a) Synapses (arrowheads) and axons (*) containing the oxidation product are darker than the rest of the tissue. Black arrows denote non-infected synapses. POm relay cell nucleus is denoted by (**). (b) High magnification of the phtooxidized synapse shown in (a). Black arrows denote non-infected synapses. Scale bars: (a) 2 µm, (b) 1 µm.

Layer 5B pyramidal (L5B) neurons of the rodent barrel cortex send a projection to the posteromedial (POm) thalamic nucleus, where they form giant synapses [21]. Here, we used the L5B-POm giant synapse of the mouse to demonstrate that also other synapses can be detected with our method (Fig. 5). Cortico-thalamic synapses were labeled with synaptophysin-EGFP and the presynaptic localization of the overexpressed protein was confirmed by co-localization with synapsin I (Fig. S6). After photooxidation, dark DAB precipitates were localized within the infected L5B-POm synapses and axonal compartments, which were readily detectable in the EM (Fig. 5a). Non-infected synapses in the same area did not contain photooxidation product and therefore appeared brighter (Fig. 5b, arrows). Despite performing the photooxidation procedure after treatment with H_2_O_2_ to block the activity of the endogenous peroxidases, mitochondria in the infected synapses are darker than the surrounding tissue, similar to the results obtained in the calyx. The EGFP-expressing synapses could be discriminated from the neighboring non-infected tissue. Axons containing EGFP and hence dark mitochondria (* in Figure 5b) could also be discriminated from the surrounding tissue. There is a difference in the intensity of the oxidation product between infected axons and infected axonal terminals, due to the fact that synaptophyin-EGFP positive structures are targeted to the presynaptic terminal.

### Distribution of the DAB Precipitate within Brain Slices

**Figure 6:**
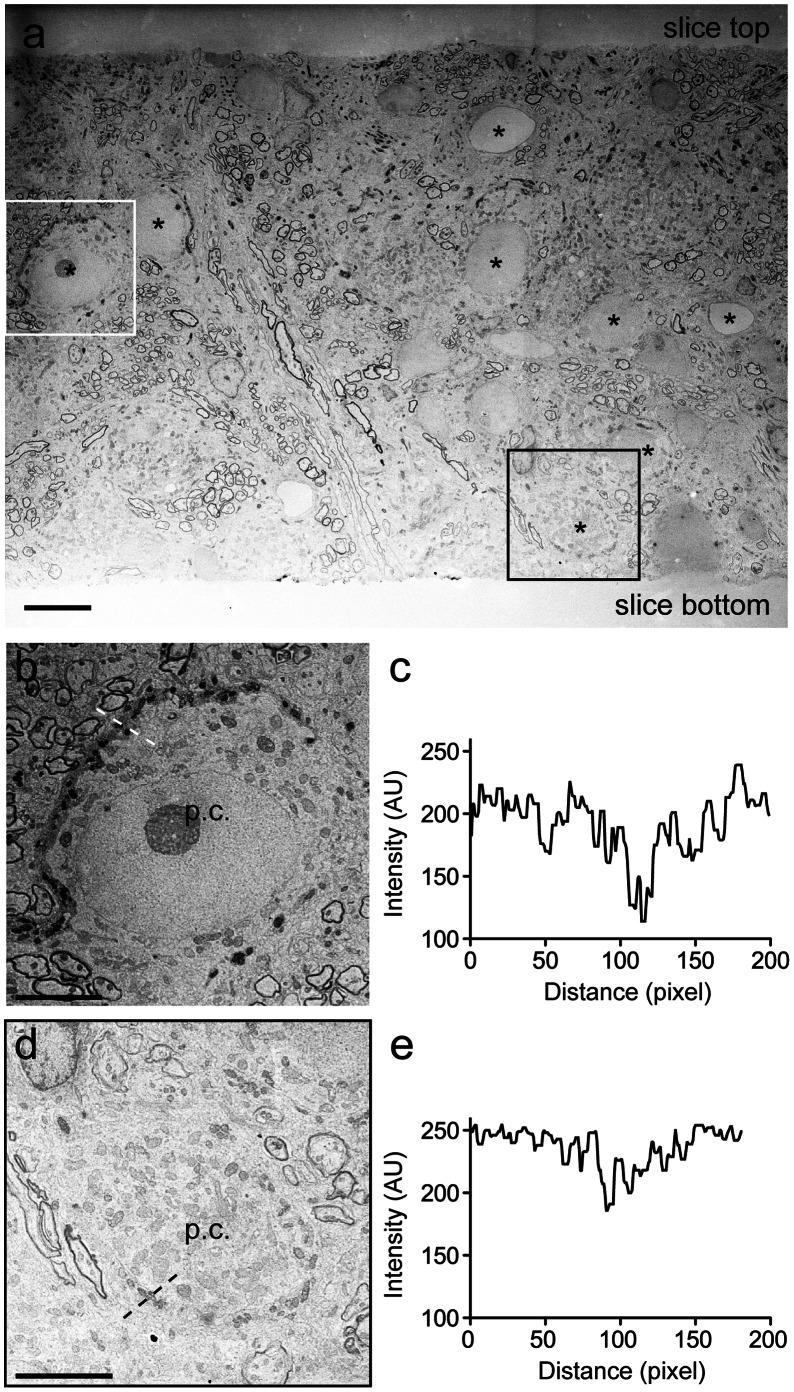
Distribution of the DAB precipitate within a 100 µm thick slice. (a) Decreasing intensity of the precipitate with increasing slice depth. A tissue slice re-sliced after photooxidation along the z-axis of the original cutting plane. Principal cell nuclei are denoted by (*). Boxes indicate digitally magnified regions shown in b and d. (b) Principal cell and calyx located in upper half of the slice (see (a)). Calyx clearly delineated by DAB precipitates. (c) Line probe showing the intensity profile of the photooxidation product within the calyx dotted line in (b). (d) Principal cell and calyx located in the lower part of the slice. Calyx contains less dense DAB precipitate (see (a)). (e) Intensity profile of the DAB oxidation product within the calyx shown dotted line in (d). p.c. MNTB principal cell. Scale bars, (a) 10 µm, (b, c) 5 µm.

To assess the distribution of the DAB precipitate along the axial direction of illumination, we re-sliced a 100 µm thick slice after photooxidation (Fig. 6). The upper side of the slice faced the illumination source rendering a presumed gradient of light intensity towards the lower surface. Correspondingly, an intensity gradient of the DAB precipitate is evident with higher intensities at the top surface and lower intensities at the bottom surface (Fig. 6a). While a MNTB principal cell localized in the upper half of the slice contains strong dark precipitates in the associated calyx of Held (Fig. 6b,c), the intensity of the DAB precipitate in another presynaptic terminal, close to the bottom of the slice is weaker but still readily discernible (Fig. 6d,e). This seems to go along with a corresponding gradient of the background intensity, resulting in a similar signal to noise ratio at different depths of the section. We typically observed a strong increase in grey scale density at mitochondria residing within the compartment expressing synaptophysin-2pHluorin, but not in mitochondria of the adjacent principal cell (Fig. 6b). This can also be seen in the weakly stained calyces at the bottom of the slice (Fig. 6d), where it can serve as a diagnostic feature. A gradient caused by graded expression levels of synaptophysin-2pHluorin cannot be excluded but is not evident in confocal image stacks (data not shown). Thus, the approach introduced here can identify DAB-positive compartments throughout the volume of 100 µm thick brain slices.

## Discussion

In this report we describe a method for the identification of GFP-labeled cellular compartments via photooxidation and subsequent serial section electron scanning microscopy of the corresponding DAB precipitates in PFA-fixed tissue of the rodent central nervous system. We show that exposure of pHluorin or EGFP to an oxygen-rich environment in an enclosed system is sufficient for the production of detectable electron-dense DAB precipitates in the labeled cells without occluding their ultrastructure. Our improved photooxidation approach allows the identification and ultrastructural characterization of neurons and their compartments expressing pHluorin, EGFP or fusion proteins thereof. However, the method cannot resolve the subcellular localization of GFP fusion proteins. In summary, the method described here allows to identify fluorescently labeled presynaptic terminals with electron microscopy.

We used 4% paraformaldehyde (PFA) in phosphate buffer saline (PBS) for tissue fixation in order to preserve the EGFP fluorescence together with the structure of the presynaptic terminals. Glutaraldehyde, which induces autofluorecence and increases unspecific background in the tissue, was excluded from the fixation procedure. This allowed us to do the correlation between EGFP signal, DAB precipitates and the EM images. Using 4% PFA we spared the application of glycine, ammonium chloride and sodium borohydrate, needed to reduce the glutaraldehyde autofluorescence [8].

We show that GFP derivatives can be used as donors of reactive oxygen species in brain tissue slices obtained from two different brain regions (brain stem and thalamus) and two species (mouse and rat) without the need of exogenous factors other than oxygen. The underlying photooxidation reaction takes advantage of free oxygen radicals that form upon illumination of the fluorophores in a tightly controlled oxygen-rich atmosphere and leads to the oxidation of DAB. The formation of the DAB precipitate depends critically on the oxygen supply to the tissue samples. Therefore, an oxygen-enriched atmosphere was created in the incubation chamber [22] at a constant temperature of 4°C.

The oxidized DAB forms networks that appear as fine granular, electron-dense precipitates at the sites of former fluorescent signals (Fig. 1), visible in the electron microscope. The precipitates were detected within the presynaptic terminal (Fig. 2 and 3). These results may appear inconsistent with the observation that GFP produces insufficient amounts of reactive oxygen-mediated DAB precipitates due to its extremely low quantum yield [9], [11]. However, our data clearly demonstrate that despite a low quantum yield EGFP can be successfully used to generate DAB precipitates when adhering to an optimized procedure including a tightly controlled oxygen-rich environment. Unfortunately, even high levels of soluble EGFP expression result in the formation of only a weak DAB photooxidation product. This might be due to a lower number of GFP molecules per volume in solution compared to GFP targeted to an intracellular organelle. The intensity of the DAB precipitation product depends on the concentration of the fluorophore within the cellular compartment. Therefore, cells expressing fluorescent proteins at low levels might remain undetected. While virus-mediated gene expression promotes high protein levels, mouse lines with GFP-labeled cells may accumulate too little protein expression to be detected. We did not probe this problem because generalizations regarding the prospects of detecting a DAB signal in a given mouse line entirely depends on the specifics of GFP expression and needs to be tested in every single case.

In contrast to previous studies relying on GFP or miniSOG fusion proteins forming assemblies of high local density [8], [9], our data show that GFP fusion proteins or soluble EGFP upon viral expression can be readily detected. miniSOG on its own shows weaker fluorescence and therefore is more difficult to detect than GFP [9], [23]. Alternatively, an ascorbate peroxidase (APEX) could be used as a genetically encoded reporter of fusion proteins for EM without the application of light [23]. However, fusion to a fluorescent protein is necessary for correlated light microscopy and EM.

Consistent with published results [4], [9], [24] we found that mitochondria in compartments expressing GFP were darker than in non-infected cells after illumination and GFP photooxidation (Fig. 4e). This might be an unspecific reaction, although addition of KCN (to inhibit mitochondrial cytochrome c oxidase) or aminotriazole (to inhibit endogenous catalase activity) provided no improvement (data not shown and Figure 5 in [9]). Nevertheless, the increased contrast of mitochondria caused by the photooxidation reaction may effectively serve as a proxy for the recognition of GFP-positive cells [4]. Only mitochondria in infected terminals were stained dark upon illumination (Fig. 4e), while those in non-infected calyces remained comparable to the mitochondria in the principal cell (Fig. S1 d,e).

Photooxidation of GFP as described here is advantageous over conventional labeling approaches such as immunogold staining, because the protein of interest is tagged before the fixation and all subsequent components are small molecules that easily penetrate tissues (O_2_, OsO_4_, DAB). This method solves multiple problems of immunohistochemical electron microscopy such as inaccessibility or destruction of antigens, lack of suitable antibodies or non-specific binding of the antibodies. Furthermore, correlative methods often require additional procedures or highly specialized instrumentation [3], [25], [26]. Using S^3^EM at a lateral resolution of 3.7 nm [15] we could trace cells containing specific reporters through a large number of ultrathin serial sections resulting in the three-dimensional reconstruction of large segments of the presynaptic terminal. The ultrastructure of the infected calyces is well-preserved and presynaptic components such as active zones and vesicle clusters could be readily identified (Fig. 4). Hence, despite the contrast added by the DAB precipitate, the ultrastructure of the labeled cells can be examined in detail without a trade-off.

The method presented here allows for the isolation of specific, genetically labeled cell populations and their examination on the ultrastructural level opening a new venue for studying specific genetically manipulated cellular assemblies, multiple cellular processes and protein distribution in intact tissue under different conditions.

## Materials and Methods

### Plasmid Construction and Viral Production

A cDNA encoding synaptophysin with two pHluorins inserted into the second intravesicular loop [18] was obtained from L. Lagnado and cloned into an adeno-associated virus (pAM-AAV2) backbone [27], [28] via insertion of a multiple cloning site containing recognition sites for XhoI and XbaI/SpeI restriction endonucleases. The synaptophysin-EGFP, EGFP and EGFP-synapsin I constructs have been described elsewhere [21], [29], [30]. The recombinant proteins were expressed under the control of chicken β-actin promoter. Recombinant chimeric viral particles containing a 1∶1 ratio of AAV1 and AAV2 were generated as described before [31], [32].

### Stereotaxic Injection and Perfusion

Experiments were conducted in accordance with the German animal welfare guidelines and were approved by the responsible authority (Regierungspräsidium Karlsruhe). Sprague Dawley rats were injected at postnatal day (P) 6 with rAAV1/2-particles encoding the protein of interest, similarly to previous reports [33], but using isoflurane anesthesia [31], [32]. Initially rats were given 5% isofluorane/O_2_, which was reduced to 1–1.5% during the surgery. Local anesthesia was maintained via subcutaneous application of approximately 50 µl lidocaine. For injection in the ventral cochlear nucleus approximately 2 µl viral solution was evenly distributed among six injection sites with the following coordinates relative to bregma and midline (in mm): (1) −9.0, 1.05; (2) −8.7, 1.05; (3) −8.4, 1.0; (4) −9.1, 0.9; (5) −8.8, 0.1; (6) −8.5, 0.9. At each of these positions injections were done at depth (z) of −0.45 ventral from bregma and axially (A_x_) at 9.5 ventral from bregma. For labeling cortico-thalamic synapses AAV1/2-synaptophysin-EGFP was injected into P14 C57BL6 mice. The following coordinates relative to bregma and midline (in mm) were used: (1) 0.0, 2.9; (2) −0.3, 2.9; (3) −0.6, 2.9; (4) −0.9, 2.8; (5) −1.2, 2.8; (6) −1.0, 2.7; (7) −0.5, 2.7. At each of these positions injections were done at depth (z) from −1.05 to 0.9 ventral from dura. 14 days after injection the animals were transcardially perfused with phosphate buffered saline (PBS) (Bioline) and fixed with 4% paraformaldehyde (PFA) in PBS, pH 7.4. Brains were immediately extracted and postfixed in 4% PFA in PBS overnight at 4°C. After fixation 100 µm-thick sections, containing the brain regions infected with rAAV, were cut on a vibratome slicer HR2 (Sigmann Elektronik, Hüffenhardt, Germany).

### Light Microscopy

Fluorescence images of sections containing infected and photooxidized calyces were acquired on a Zeiss Axiophot microscope equipped with a FluoArc fluorescent lamp and INTAS Camera (INTAS Science Imaging, Göttingen, Germany). A 40x oil-immersion Plan-NEOFLUAR objective N.A. 1.3 was used. Images were obtained with QCapture software (Quantitative Imaging Corporation, Canada).

### Photooxidation

Brain regions containing infected presynaptic terminals (medial nucleus of the trapezoid body or posteromedial thalamic nucleus) were washed 3x in cold phosphate buffer saline (PBS), followed by 3x in cold 50 mM Tris-HCl pH 7.6. Sections were oxygenized via a frit device (air stone) for 60 min with pure medical O_2_ in 50 mM Tris-HCl pH 7.6 at 4°C and stored overnight in a tightly closed system, e.g. conical centrifuge tube (50 ml). The volume of the closed container was filled with oxygen. On the following day the sections were again exposed to pure O_2_ in 50 mM Tris-HCl pH 7.6 for 60 min at 4°C followed by 10 min incubation in 25 ml ice-cold, oxygenized 50 mM Tris-HCl pH 7.6 solution containing 25 mg 3,3′-Diaminobenzidine (DAB) (concentration 1 mg/ml, Serva Electrophoresis, Heidelberg Germany) and 125 µl 1 M NaOH (final concentration 5 mM). After 3–5 min incubation period the sections were transferred to a custom-made metal chamber attached to an external cooling device (ice box with a pumping unit) to maintain constant temperature of 4°C (adapted from [22]). The DAB-containing solution was poured into the chamber and the slice was placed in it. Samples were immobilized on a cover slip (Menzel-Gläser; grade 00; thickness 108 µm) with a nylon net (200×200 µm) attached to a platinum ring.

The cooling chamber was positioned on the microscope stage and was attached to the external cooling device. An inverted microscope (Leica DM IRBE) equipped with a 40x oil immersion objective (NA: 0.75–1.25) and a 100 W mercury discharge lamp was used for the photooxidation. The lamp was set to 50% of its maximum intensity. The objective was positioned above the region of interest and the light path aperture was closed to form a spot with a diameter of 100–200 µm. The region of interest was illuminated with an excitation wavelength of 488 nm at maximum intensity for 5 min with NA 0.75, followed by 5 min with NA 1.0 and then by 5 min with NA 1.25. This procedure was found to result in the highest efficiency of photooxidation. Formation of fine precipitates was observed in the exposed area after 10–12 min. After 15 min light exposure the illumination was stopped and the reaction was terminated.

### Controls

Non-infected MNTBs were used as an internal control for the formation of DAB precipitates. Control experiments were performed under the same illumination conditions (15 min, λ = 488 nm, decreasing NA) as the GFP-expressing terminals. No reaction product was observed in this case (Fig. S1). The photooxidation in the thalamic POm nucleus was performed after treatment with H_2_O_2_ to block the endogenous peroxidases, so that the illuminated fluorescent protein was the only source for DAB photooxidation (Fig. 5). The results were comparable to conditions omitting these agents.

### Tissue Preparation and Osmification

After photooxidation the tissue was washed 3x with 50 mM Tris-HCl pH 7.6 and with 0.1 M cacodylic acid buffer pH 7.4. The MNTB area containing the precipitate was dissected out with a scalpel in such a way that the slice surface facing the objective is easily identifiable. The tissue was post-fixed in a mixture of 2% osmiumtetroxide and 1.5% potassium ferrocyanide in distilled water (dH_2_O) for 60 min at RT. After 3 washing steps in dH_2_O (total washing time 1–2 h) the tissue was dehydrated in an ascending series of alcohol followed by propylene oxide and stored for 3 h at RT in a mixture consisting of propylene oxide and epoxy (Serva Electrophoresis, Heidelberg, Germany) (1∶1).

### Embedding

After infiltration with the epoxy resin the slice was embedded between two microscopy slides coated with Teflon paste (Polyscience) and polymerized at 60°C for 36 h. After polymerization, the microscopy slides were removed and the tissue slice was mounted onto a pre-polymerized epoxy block with the side, previously faced by the objective pointing now upwards.

### Block Preparation and Serial Sectioning

Once the resin was firm, the block was trimmed to a rectangular shape as previously described [34] using a 35° diamond trimmer (Diatome, Switzerland). We used an ultramicrotome (UltraCut S, Reichert, Vienna, Austria) to cut serial sections. Initially, 50 nm-thick sections were cut until the region of interest was reached. The sections were placed onto a hydrophilic wafer strip for visual inspection. DAB precipitates were easily identified in non-counterstained sections by scanning electron microscopy and a direct correlation between light microscopy and EM was performed at 3,000×. After identifying the region of interest 16–17 serial sections per sample with a thickness of approx. 35 nm were cut.

Low magnification images were used to locate the infected and photooxidized terminals. Only then, the ribbons were prepared and the first sections containing the overview images were lost during the slicing procedure. Then high-resolution images were obtained from regions that are located deeper in the original slice (approx. 35–70 nm below the slice surface) than the overview images. Hence the 3D reconstructions were done from consecutive images different from the original overview. Therefore, deviations between the overview images and the high-resolution images are possible. This is also true for the images obtained before and after counterstaining (Fig. S2). The intensity difference between the oxidized product and the rest is most prominent at low magnification (Figs. 2 and 3).

### Preparation of the Silicon Wafer Strip

The ribbon was taken up with a custom made device attached to ultramicrotome [15]. The silicon wafer (Si-Mat, Silicon Materials, Germany) was cleaned and made hydrophilic via incubation in sulfuric acid and perhydrol (1∶1) for 120 min followed by 3 rinses in distilled water. Once the ribbon was attached to the wafer, the wafer was slowly removed and allowed to air dry. Overview images and images of single cells were acquired on unstained tissue to prevent false positives. After that sections were counterstained in saturated solution of uranyl acetate in dH_2_O for 17 min, washed 3× in dH_2_O and exposed for 7 min to Reynolds Lead citrate in CO_2_ free atmosphere. At the end the wafer was rinsed with dH_2_O and air-dried. High-magnification images of selected presynaptic segments were obtained from the stained tissue.

### SEM Imaging

A LEO Gemini 1530 with a FEG was used for SEM imaging [15]. Inlens-detector with a working distance of 1.8 mm, a 60 µm aperture and 3 keV acceleration voltage was used. The images were inverted to obtain images comparable to TEM. Overview images were taken at 600×magnification, images of single cells – at 3,000×magnification. Selected presynaptic segments were imaged at magnification of 10,000×. In this case an area of 11.2 µm×8.4 µm of the specimen was digitized by 3,072×2,304 pixels resulting in a pixel size of 3.87 nm×1.8 nm. The pixel dwell time was 40 µs. Pre-alignment was done manually on the SEM through correlating the previous image with the next by stage rotation and shifting of the electron beam. Images were acquired once before counterstaining and once afterwards.

### Data Analysis

Images were corrected for background signal using Adobe Photoshop CS4, Version 11.0.2. In the light microscopic images of photooxidation products the background was set to maximum values and the photooxidation product – to minimum values. 3D analysis of the data was made by alignment of the serial sections using OpenCAR software, version 1.5.79 available from http://opencar.ulster.ac.uk as previously described [15]. Visualization of 3D data was performed with AMIRA 5.3.1 software (Visage Imaging, Berlin, Germany). Final images were aligned in Adobe Illustrator CS4, Version 14.0.0.

## Supporting Information

Figure S1 Mitochondria in non-infected presynaptic terminals remain unchanged. (a) Overview of the MNTB showing a principal cell (p.c.) and associated calyx of Held terminal. Images are obtained without the application of contrasting agents and cells appear fuzzy due to the presence of DAB. (b, c) Images with higher resolution of the same calyx as in (a) show mitochondria and synaptic vesicles within the presynaptic plasma membrane. Black arrows point out the presynaptic compartment. (d, e) Digital magnifications of the presynaptic compartments, showing the absence of DAB precipitate within the terminals. Mitochondria in both presynaptic and postsynaptic compartments show comparable intensity (insets). Intensity (in arbitrary units (AU)) is plotted along the Y-axis. Scale bars, (a) 2 µm; (b, c) 1 µm; (d, e) 500 nm.
(TIF)

Figure S2 DAB precipitates form in fluorescently labeled presynaptic terminals only within the region of illumination. (a) A fluorescence microscopy overview of an infected MNTB after illumination. Photooxidation results in the weakening of the fluorescent signal and the labeled calyces are not visible. (b) Low resolution EM image of a MNTB segment obtained from the rim of the illuminated region. A presynaptic terminal, which was inside the illuminated region, contains DAB precipitate (arrows). A neighboring synapse, which remained outside the illuminated region, does not show visible precipitate. (c) Digital magnification of the principal cell and the presynaptic terminal, denoted by (*) in (b). (d) Digital magnification of the principal cell and the calyx, denoted by (**) in (b). p.c. MNTB principal cell. Scale bars, (a) 100 µm, (b, c, d) 10 µm.
(TIF)

Figure S3 Photooxidized DAB precipitates are visible prior to counterstaining with uranyl acetate. Cells #10/11 prior to (a) and after (b) incubation in uranyl acetate. Cell #8 prior to (c) and after (d) incubation in uranyl acetate. White arrows indicate dark precipitates within the infected terminals. Scale bars 15 µm.
(TIF)

Figure S4 An additional example of a photooxidized calyx at higher magnification. (a) Cell #2 shown in Figure 1 imaged at a low magnification. Principal cell nuclei are denoted by (*). (b) High magnification image of the presynaptic segment pointed out with arrows in (a). (c) Corresponding 3D reconstruction generated from 16 consecutive sections (35 nm thickness). (c-1) Digitally magnified segments from the presynaptic compartments, corresponding to the rectangular shape in (c-1) Presynaptic segment – white, synaptic vesicles – red spheres, mitochondria – cyan, p.c. – principal cell. Scale bars, (a) 5 µm; (b) 2 µm.
(TIF)

Figure S5 Photooxidation of soluble EGFP and EGFP-synapsin I in the calyx of Held. Dark spots, containing DAB precipitates (arrows), were successfully detected along the circumference of the MNTB principal cell in putative presynaptic compartments expressing soluble EGFP (a) and EGFP-synapsin I (b) ten days after infection using the described method. (c) High magnification image obtained from a terminal containing EGFP. (d) High magnification image from a terminal infected with EGFP-synapsin Ia. (e) Digital magnification of the boxed region in (c). (f) Digital magnification of the boxed region in (b). Scale bars, (a,b) 10 µm; (c,d) 2 µm; (e,f) 1 µm, p.c. – principal cell.
(TIF)

Figure S6 Colocalization of synaptophysin-EGFP and synapsin in corticothalamic synapses. (a) Expression pattern of synaptophysin-EGFP in the POm, ten days after injection. (b) Synapses labeled with antibodies against the presynaptic protein synapsin I. (c) Co-localization of the two signals shows infected (arrows) and non-infected (*) giant synapses. The POm relay cells (r.c.) are discernable as a dark spots without any fluorescent signal embedded in the synapsin I positive neuropil. Images represent a single confocal plane obtained on Leica SP5 with a 63x glycerol-immersion objective and 5x digital zoom. (d) Schematic drawing of POm relay cells shown in (c) (black square). Giant synapses (green – synaptophysin-EGFP positive synapses, red – synaptophysin-EGFP negative synapse) coming from L5B pyramidal cells are situated primarily on the soma and proximal dendrites of the thalamic cell. The relay cells receive also small modulatory synapses (blue) on their distal dendrites. Scale bar, 10 µm.
(TIF)

Movie S1 Raw data used for the three-dimensional reconstruction presented in Fig. 3e.
(AVI)

Movie S2 Raw data used for the three-dimensional reconstruction presented in Fig. 3f.
(AVI)

Movie S3 Raw data used for the three-dimensional reconstruction presented in Fig. S4.(TIF)
(AVI)
