# Supplementary figures and images for: Photooxidation-Guided Ultrastructural Identification and Analysis of Cells in Neuronal Tissue Labeled with Green Fluorescent Protein
Source: PLoS One. 2013 May 31;8(5):e64764. doi: 10.1371/journal.pone.0064764 (PMC3669359; doi:10.1371/journal.pone.0064764)

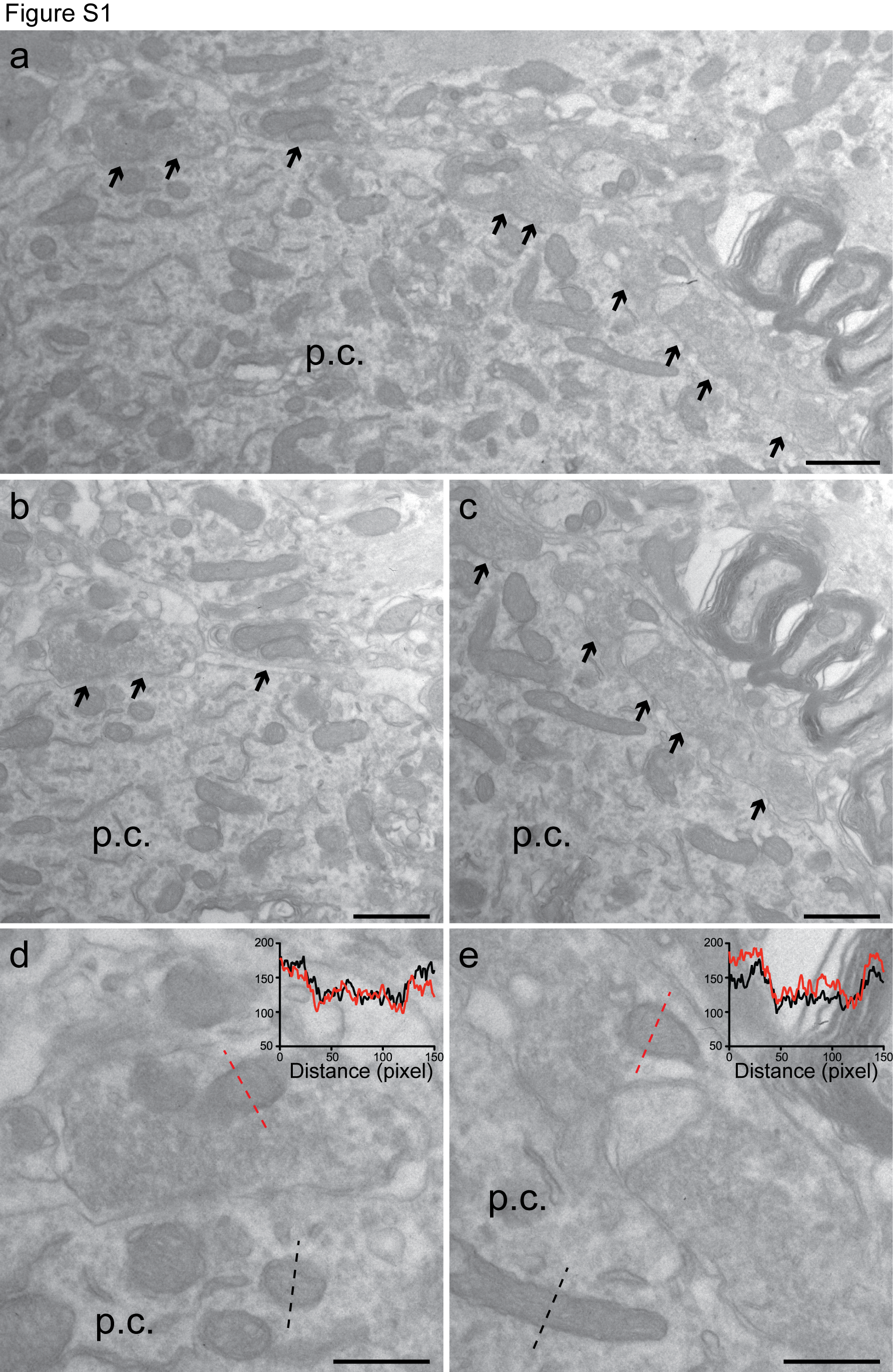

Supplement: Figure S1 — Mitochondria in non-infected presynaptic terminals remain unchanged. (a) Overview of the MNTB showing a principal cell (p.c.) and associated calyx of Held terminal. Images are obtained without the application of contrasting agents and cells appear fuzzy due to the presence of DAB. (b, c) Images with higher resolution of the same calyx as in (a) show mitochondria and synaptic vesicles within the presynaptic plasma membrane. Black arrows point out the presynaptic compartment. (d, e) Digital magnifications of the presynaptic compartments, showing the absence of DAB precipitate within the terminals. Mitochondria in both presynaptic and postsynaptic compartments show comparable intensity (insets). Intensity (in arbitrary units (AU)) is plotted along the Y-axis. Scale bars, (a) 2 µm; (b, c) 1 µm; (d, e) 500 nm. (TIF) [file pone.0064764.s001.tif]

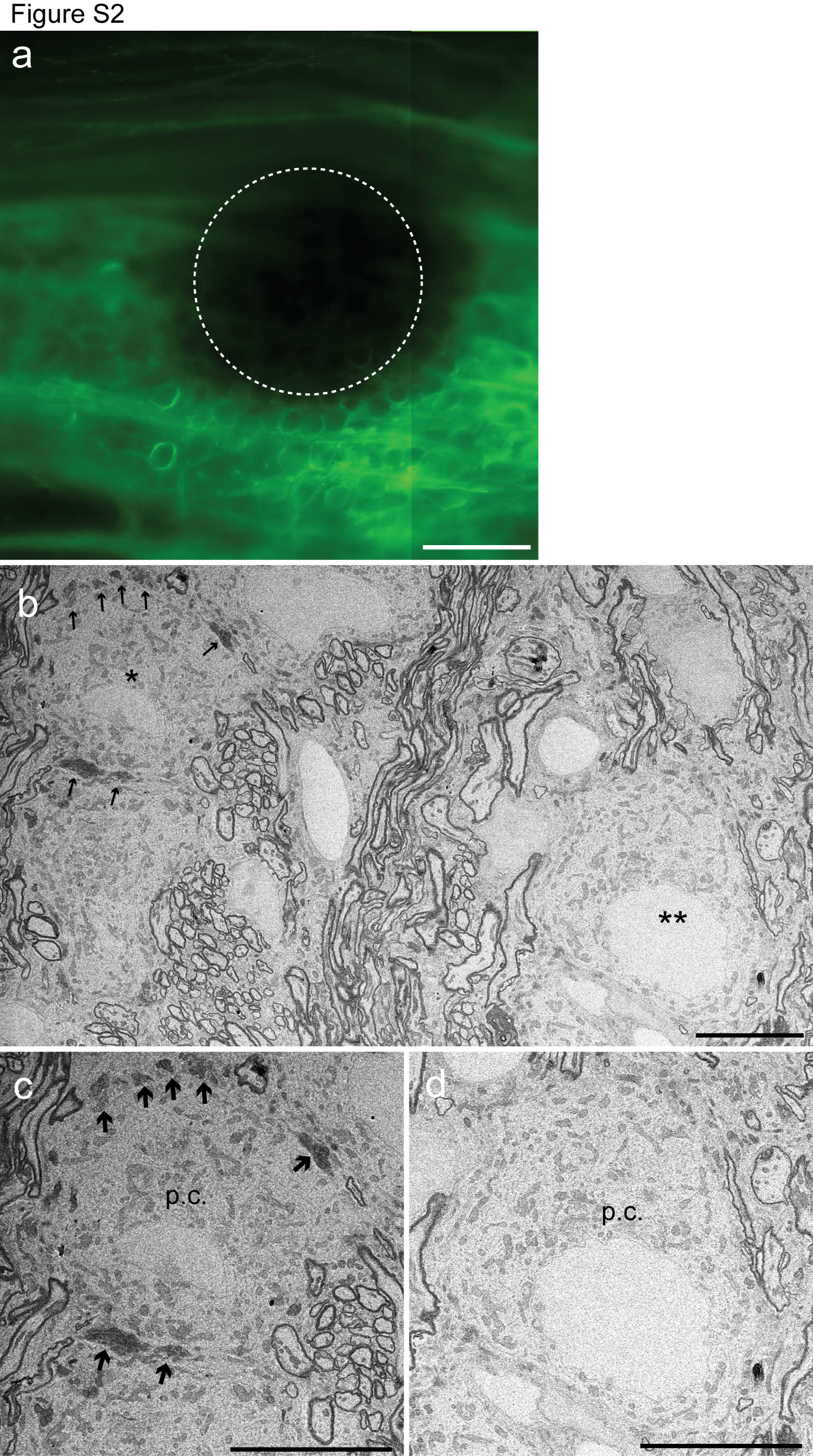

Supplement: Figure S2 — DAB precipitates form in fluorescently labeled presynaptic terminals only within the region of illumination. (a) A fluorescence microscopy overview of an infected MNTB after illumination. Photooxidation results in the weakening of the fluorescent signal and the labeled calyces are not visible. (b) Low resolution EM image of a MNTB segment obtained from the rim of the illuminated region. A presynaptic terminal, which was inside the illuminated region, contains DAB precipitate (arrows). A neighboring synapse, which remained outside the illuminated region, does not show visible precipitate. (c) Digital magnification of the principal cell and the presynaptic terminal, denoted by (*) in (b). (d) Digital magnification of the principal cell and the calyx, denoted by (**) in (b). p.c. MNTB principal cell. Scale bars, (a) 100 µm, (b, c, d) 10 µm. (TIF) [file pone.0064764.s002.tif]

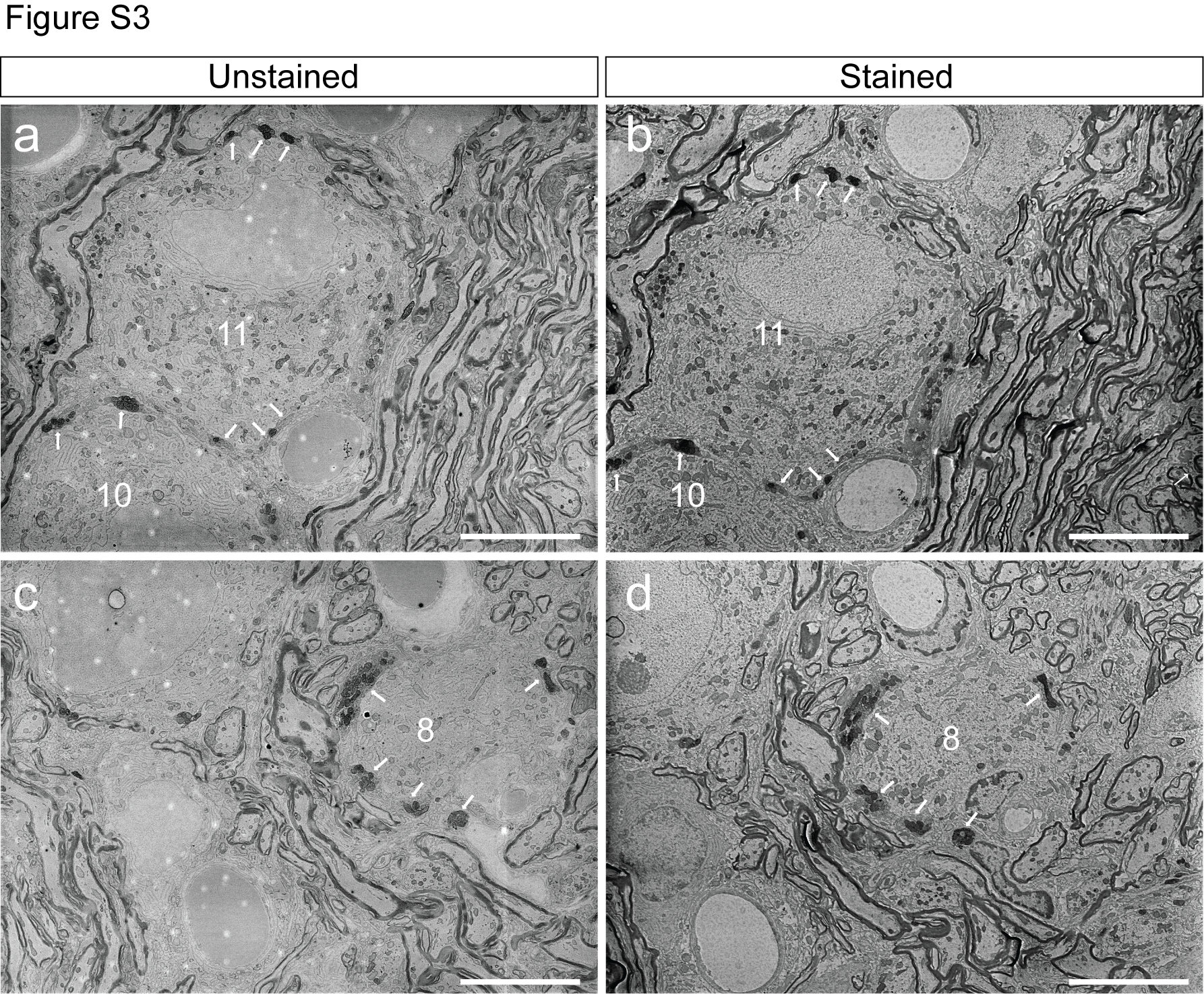

Supplement: Figure S3 — Photooxidized DAB precipitates are visible prior to counterstaining with uranyl acetate. Cells #10/11 prior to (a) and after (b) incubation in uranyl acetate. Cell #8 prior to (c) and after (d) incubation in uranyl acetate. White arrows indicate dark precipitates within the infected terminals. Scale bars 15 µm. (TIF) [file pone.0064764.s003.tif]

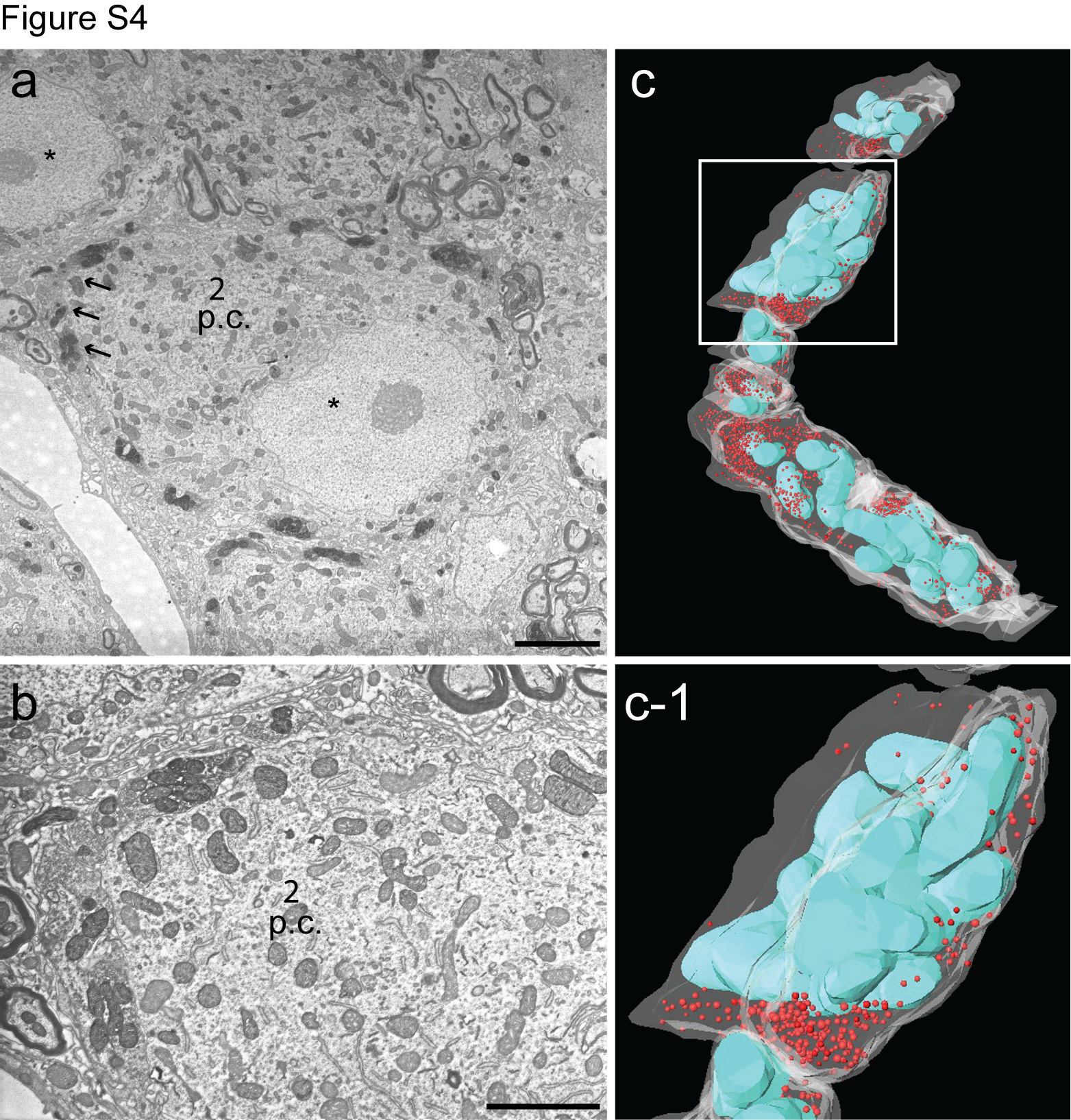

Supplement: Figure S4 — An additional example of a photooxidized calyx at higher magnification. (a) Cell #2 shown in Figure 1 imaged at a low magnification. Principal cell nuclei are denoted by (*). (b) High magnification image of the presynaptic segment pointed out with arrows in (a). (c) Corresponding 3D reconstruction generated from 16 consecutive sections (35 nm thickness). (c-1) Digitally magnified segments from the presynaptic compartments, corresponding to the rectangular shape in (c-1) Presynaptic segment – white, synaptic vesicles – red spheres, mitochondria – cyan, p.c. – principal cell. Scale bars, (a) 5 µm; (b) 2 µm. (TIF) [file pone.0064764.s004.tif]

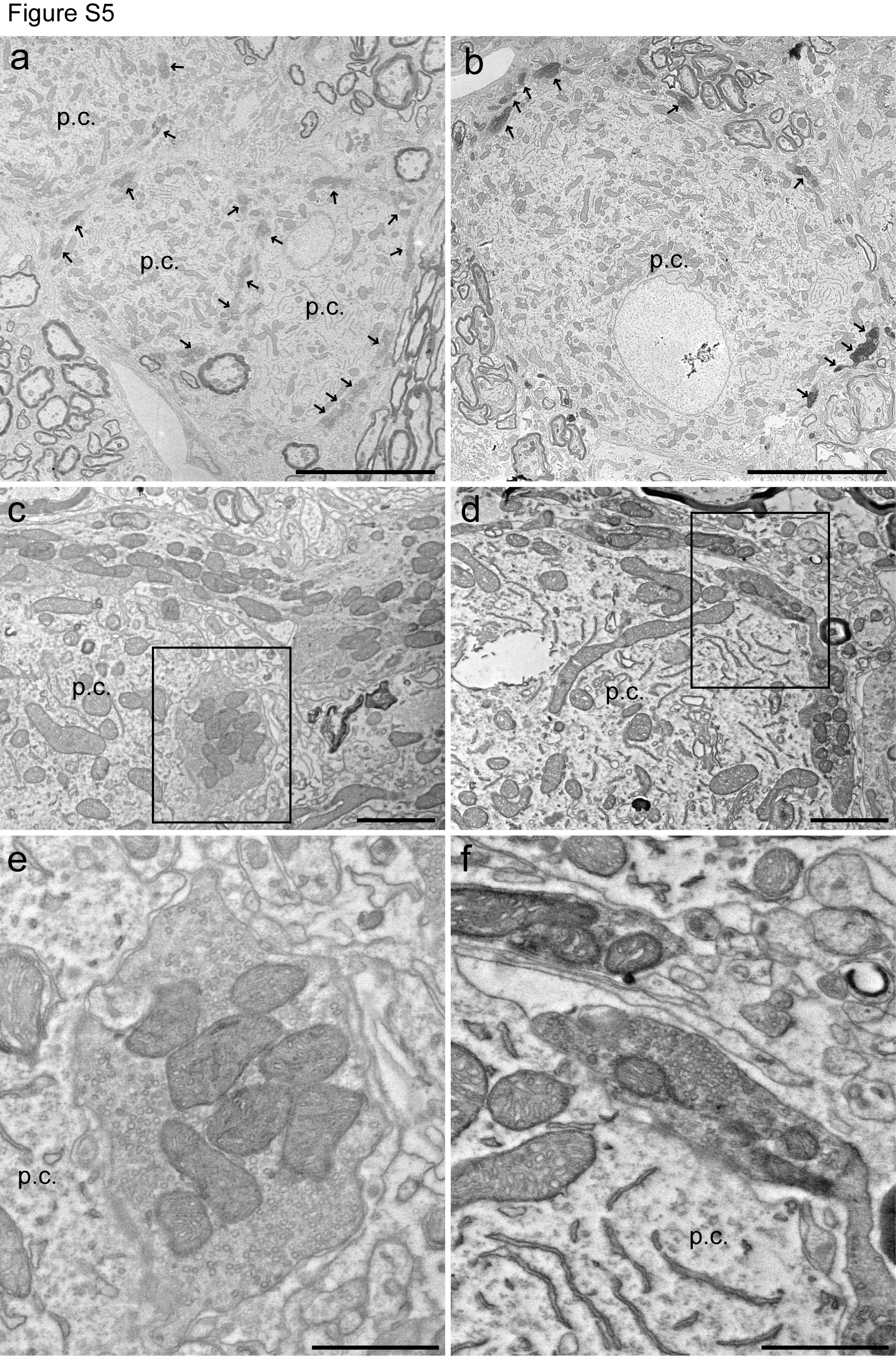

Supplement: Figure S5 — Photooxidation of soluble EGFP and EGFP-synapsin I in the calyx of Held. Dark spots, containing DAB precipitates (arrows), were successfully detected along the circumference of the MNTB principal cell in putative presynaptic compartments expressing soluble EGFP (a) and EGFP-synapsin I (b) ten days after infection using the described method. (c) High magnification image obtained from a terminal containing EGFP. (d) High magnification image from a terminal infected with EGFP-synapsin Ia. (e) Digital magnification of the boxed region in (c). (f) Digital magnification of the boxed region in (b). Scale bars, (a,b) 10 µm; (c,d) 2 µm; (e,f) 1 µm, p.c. – principal cell. (TIF) [file pone.0064764.s005.tif]

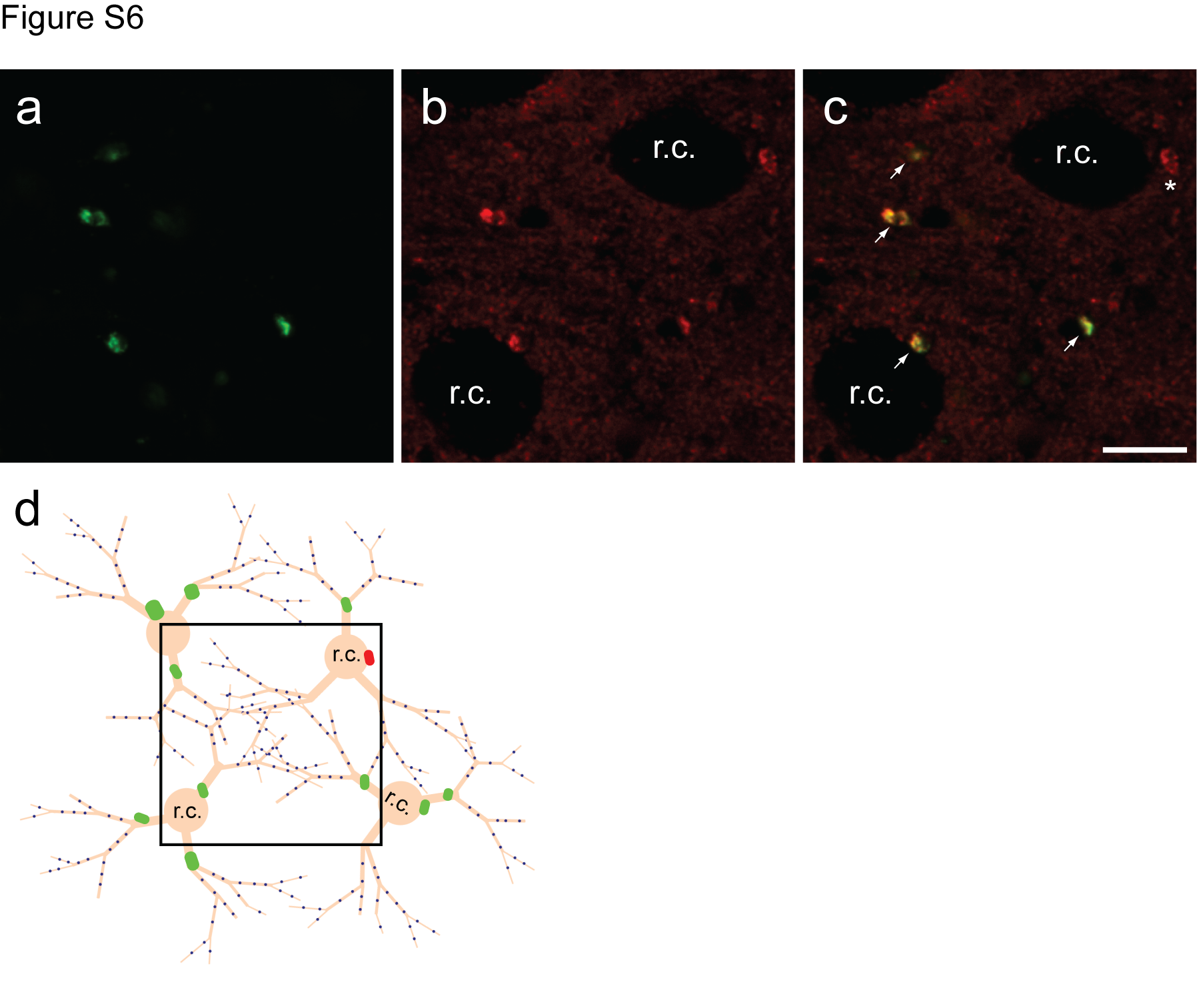

Supplement: Figure S6 — Colocalization of synaptophysin-EGFP and synapsin in corticothalamic synapses. (a) Expression pattern of synaptophysin-EGFP in the POm, ten days after injection. (b) Synapses labeled with antibodies against the presynaptic protein synapsin I. (c) Co-localization of the two signals shows infected (arrows) and non-infected (*) giant synapses. The POm relay cells (r.c.) are discernable as a dark spots without any fluorescent signal embedded in the synapsin I positive neuropil. Images represent a single confocal plane obtained on Leica SP5 with a 63x glycerol-immersion objective and 5x digital zoom. (d) Schematic drawing of POm relay cells shown in (c) (black square). Giant synapses (green – synaptophysin-EGFP positive synapses, red – synaptophysin-EGFP negative synapse) coming from L5B pyramidal cells are situated primarily on the soma and proximal dendrites of the thalamic cell. The relay cells receive also small modulatory synapses (blue) on their distal dendrites. Scale bar, 10 µm. (TIF) [file pone.0064764.s006.tif]
